# Notes and News

**Published:** 1897-09-04

**Authors:** 


### Notes and News.

(Collected and Communicated.)

Diphtheria has been very prevalent in the metropolis during the present summer.

The plague is largely present in Poona. Due precautions against its spread are being taken, and the bazaar has been closed.

Some little time past we commented upon the theft of a large number of inoculated rabbits from a bacteriological laboratory in Paris. The thieves have been discovered, and each sentenced to imprisonment. Those persons who are known to have eaten the rabbits experienced no ill effects.

A working carpenter at Chertsey has bequeathed £773 to the County Hospital, Guildford, out of hard-earned savings. Such a gift is very encouraging. It shows not only the value which the working man now attaches to our hospitals, but also it is possible for a working man to lay by a considerable sum if he chooses.

Cleaning and repairing is going on at a large number of the London hospitals, and some institutions have been entirely closed for a short time. Although this proceeding may send extra work elsewhere, there is no doubt that an absence of patients, and the consequent sweetening of the entire building, is a very excellent thing.

Considerable disturbances have taken place at the Mercers' Hospital, Dublin. The medical staff have been dismissed by the governors, and a new one elected. A member of the former staff, it is stated, has continued to visit some of his patients, and several of these were discharged by the House Committee, but as they did not leave it is stated that they were left without food.

The proposed convalescent home scheme for Bristol gives another example of the tendency of a few generous persons to give very largely, whilst the co-operation of every inhabitant who could give is not attained as it should be. At Bristol the influx of contributions towards the new home languished. Mr. J. S. Fry at once came forward and increased his offer of £1,000 to £10,000, provided the required sum is forthcoming at the end of the year, and Sir W. Wills has offered £5,000 on the same condition. Thirteen thousand pounds is now required.

The sixty-fifth annual meeting of the British Medical Association was inaugurated at Toronto on August 30th. Dr. Roddick, Professor of Surgery in the McGill University, the President of the Association, delivered the opening address. The Governor-General was present. The social programme is very full and elaborate. A museum was opened in honour of the occasion.

Princess Henry of Battenberg visited Ryde on the 30th ult. and laid the foundation-stone of the new children's wing at the Royal Isle of Wight Infirmary. The wing is to be a souvenir of the Queen's long reign. £6,000 has been subscribed for the purpose by 1,500 subscribers. Ten cots will be provided, two of which are endowed.

Professor Gokhlee has withdrawn the charges against the Poona Plague Committee, and fully apologised. Professor Gokhlee says that his bitterest regret is that he has added to the terrible anxieties of the Governor, and that he has been less than just to the English soldiers, and had made grave, unwarranted charges against them. He tenders an unqualified apology to all concerned.

In connection with the opening of the medical schools in October, addresses will be delivered at St. George's Hospital by Dr. Patrick Manson; at St. Mary's Hospital, by Dr. Gow; at Charing Cross, by Dr. William Carter, Professor of Materia Medica, Liverpool; at the Middlesex Hospital, by Lord Strathcona and Mount Royal, Chancellor of the M'Gill University, Montreal. At most of the schools the opening ceremony will be followed by the usual dinner for past and present students.

That the Middlesex Hospital has not been forgotten at this time, when the ignorant are stating that the hospitals are suffering severely, is apparent by the statement made at the last quarterly court of the governors. Ten thousand pounds has been received in legacies; upwards of £1,600 has been received in donations, apart from the awards of the Hospital Sunday and Saturday Funds, amounting to upwards of £4,100. We are glad to learn that the beautiful convalescent home at Clacton-on-Sea proves as beneficial as was expected.

We have often pointed out that the best managed of the hospitals have always been glad to co-operate, and that inter-communication between them was a thing of every-day occurrence, and tended greatly to promote their well-being and good management. We have much pleasure in announcing the formation of a central association of representatives of London hospitals with medical schools. To commence with, nine of the large hospitals are included in the association, and power is taken to admit from time to time representatives of other hospitals on conditions to be laid down in the future. Each hospital which enters the association will appoint three representatives, one of whom will retire annually. The main object is to provide for the consideration of any matter in which the hospitals have a common interest, and to promote joint action in regard to it.

